# Research highlights, *The Plant Genome*, Volume 15, Issue 2

**DOI:** 10.1002/tpg2.20233

**Published:** 2022-06-14

## 1 WORKFLOW BOOSTS PLANT GENOME ALIGNMENT

Wu et al. (https://doi.org/10.1002/tpg2.20204) developed a practical multiple alignment workflow for plant genomes and tested a range of key parameters to improve alignment rates. They found that by tuning the repeat masking and alignment approaches, alignment rates could be substantially boosted, particularly for noncoding sequences. The alignment workflow was tested using a set of 33 grass species, three different repeat masking methods, and various parametrizations of the LAST aligner. These findings provide a new workflow for aligning plant genomes and can help align diverged noncoding sequences to better characterize plant genome evolution.

## 2 WHEAT PLOIDY MATTERS FOR FHB RESISTANCE

Fusarium head blight (FHB) is a devastating disease of bread and pasta wheat that differ at ploidy. Zhu et al. (https://doi.org/10.1002/tpg2.20183) report that FHB resistance genes act differently in bread and pasta wheat. Specific pasta wheat chromosomes were identified to contain unique genes that negatively or positively regulate the expression of bread wheat‐derived FHB resistance genes by chromosome substitution. The findings of this work will enhance the deployment of resistance genes to fight against FHB in wheat.

## 3 HISTORICAL *NEC3* DISEASE‐LESION MIMIC MUTANT CLONED IN BARLEY

Disease lesion mimic or necrotic mutants display leaf lesions in the absence of pathogen infections and are instrumental in studying host–pathogen interaction. Rosignoli et al. (https://doi.org/10.1002/tpg2.20187) describe a collection of 34 new disease lesion mimic mutants in barley and identify the gene responsible for the historical *nec3* orange/tan mutant. The gene encodes a cytochrome P450 CYP71P1 enzyme involved in serotonin synthesis, likely regulating endogenous cell oxidation and death. The gene was cloned by complementation by sequencing, a novel time and cost‐effective method, which relies on the availability of two or more mutants with the same phenotype. Phylogenetic analysis showed that *CYP71P1* emerged early in angiosperms but has been lost in some lineages including *Arabidopsis*.

## 4 COORDINATE REGULATION OF TOMATO ALKALOID BIOSYNTHESIS

Tomato consumption is associated with many health benefits including decreased risk for the development of various chronic diseases. Recent data suggests that steroidal alkaloids are a unique chemical component of tomatoes that may contribute to positive health outcomes. However, the quantity, diversity, and production of these alkaloids in tomato fruits is poorly understood. Dzakovich et al. (https://doi.org/10.1002/tpg2.20192) found that wild ancestors of tomatoes tended to have higher amounts of steroidal alkaloids in their fruits compared to modern varieties and this outcome is under strong genetic control. They also discovered a genetic region that controls the production of early pathway steroidal alkaloids, including alpha‐tomatine. These findings represent important conclusions for plant metabolism and provide genetic targets that could be used to create varieties of tomatoes to address unanswered questions about the role of tomato steroidal alkaloids in defense, flavor, and human health.

## 5 US WHEAT DIVERSITY HAS NOT DECREASED

There is an enduring concern that modern plant breeding results in loss of genetic diversity. In the case of U.S. wheat, Sthapit et al. (https://doi.org/10.1002/tpg2.20196) found that spring wheat after 1961 is very different from older varieties in terms of population structure, possibly due to the influence of germplasm and cultivars developed by the CIMMYT breeding program. However, the quantitative level of genetic diversity of single nucleotide polymorphism markers has not decreased over time for either spring or winter wheat, despite periodic fluctuations.

## 6 SWEET CORN TRANSCRIPTOME PINPOINTS VITAMIN GENES

Sweet corn is a popular vegetable in the United States with strong potential for the biofortification of vitamins that could benefit human health. Using 3′mRNA transcript abundances from fresh sweet corn kernels, Hershberger et al. (https://doi.org/10.1002/tpg2.20197) conducted transcriptome‐wide association studies and transcriptome‐ and genome‐wide marker predictions for carotenoids (provitamin A, lutein, zeaxanthin, and antioxidants) and tocochromanols (vitamin E and antioxidants). These analyses revealed associations of *β‐carotene hydroxylase* (*crtRB1*), *lycopene epsilon cyclase* (*lcyE*), *deoxy‐xylulose synthase2* (*dxs2*), *diphosphocytidyl methyl erythritol synthase2* (*dmes2*), *cytidine methyl kinase1* (*cmk1*) with carotenoid traits. Additionally, *γ‐tocopherol methyltransferase* (*vte4*), *homogentisate geranylgeranyltransferase* (*hggt1*), and *geranylgeranyl hydrogenase1* (*ggh1*) were associated with tocochromanol traits. Transcriptomic data boosted predictive ability over genome‐wide marker data alone for some traits, but joint transcriptome‐ and genome‐wide marker models achieved the highest predictive abilities. These findings will be leveraged to speed up the breeding process for developing biofortified sweet corn cultivars.

## 7 EVOLUTION OF OSCA GENES IN PLANTS

Water is vital for plants. The osmosensor OSCA1, a member of the OSCA Ca^2+^ channel family, perceives the initial water stress. Wu et al. (https://doi.org/10.1002/tpg2.20198) discovered the evolution of OSCA genes is consistent with plant transition from water to land, as the green algae is directly basal to the streptophyte clades 1–3. The streptophyte clades 1–3 present different gene expansion patterns. OSCAs in early land plants were under decelerated evolution, whereas OSCAs in seed plants showed accelerated evolution. OSCAs may have evolved to sense water stress in the ancestor of seed plants and ferns, which occupies typical leaves, typical roots and phloem tissues, all of which require osmosensors to maintain water balance and food conduction.

## 8 DIFFERENTIAL GENE EXPRESSION IN TALL FESCUE TISSUES IN RESPONSE TO WATER DEFICIT

Tall fescue is a popular pasture and turf grass particularly known for drought resistance that allows for its persistence in locations that are unfavorable for other cool‐season grasses. Crown survival under stress is critical to plant survival because it contains the apical meristems and harbors its symbiotic fungal endophyte that has been shown to contribute to persistence. In this work, Chakrabarti et al. (https://doi.org/10.1002/tpg2.20199) subjected tall fescue plants harboring their endophyte to water‐stress and compared gene expression in leaf blades, pseudostems, crowns, and roots. A number of differential pathways across all tissues included photosynthesis, carbohydrate metabolism, phytohormone biosynthesis and signaling, cellular organization, and transcriptional regulation. While no pathway was observed to be differentially expressed specifically in the stressed crown tissues, genes encoding auxin response factors, nuclear pore anchors, structural maintenance of chromosomes, and class XI myosin proteins were more highly differentially expressed in crown than in the other vegetative tissues, suggesting that regulation in expression of these genes in the crown may aid in survival of the meristems in the crown.

## 9 MULTI‐TRAIT, MULTI‐LOCUS MODELS ARE USEFUL FOR GWAS

The utility of GWAS models that consider either multiple traits or multiple loci has been demonstrated. Fernandes et al. (https://doi.org/10.1002/tpg2.20200) implemented a statistical approach called MSTEP that includes both multiple traits and multiple loci in one statistical model. Using real marker data in maize and soybean, we conducted a simulation study to evaluate the performance of MSTEP relative to two competing GWAS approaches. Although MSTEP did not uniformly perform the best across all simulations, we concluded that MSTEP is well suited to identify loci underlying low‐heritable polygenic traits, with a mixture of pleiotropic and non‐pleiotropic loci. We therefore encourage GWAS researchers to use MSTEP in addition to other existing GWAS models.

## 10 DNA METHYLATION REGULATES HYPERHYDRICITY OF IN VITRO SEEDLINGS

Hyperhydricity is the most universal physiological disorder during plant tissue culture. Gao et al. (https://doi.org/10.1002/tpg2.20202) discovered that the global methylation level decreased in hyperhydric seedlings, and most of the differentially methylated genes were CHH hypomethylated genes. Hyperhydric seedlings displayed CHH demethylation patterns in the promoter of the *ACS1* and *ETR1* genes, resulting in upregulated expression of both genes and increased ethylene accumulation. Hyperhydric seedlings also displayed reduced stomatal aperture accompanied by decreased water loss and increased phosphorylation of aquaporins accompanied by increased water uptake. DNA demethylation of ethylene‐related gene promoters may be a key switch that activates ethylene pathway genes to affect ethylene synthesis and signal transduction, which subsequently influence water metabolism, leading to hyperhydricity.

## 11 BREEDING FOR PLANT METABOLITES

Shifting plant metabolite concentrations is important for plant breeders seeking to improve crop nutrition and agronomic performance and developing metabolite selection methods that are transferable across plant populations would benefit plant breeding programs. Brzozowski et al. (https://doi.org/10.1002/tpg2.20205) report methods for genomic prediction of more than 600 metabolites in oat seeds across spring oat populations. After genetically characterizing metabolites in a diverse oat panel, they used multikernel genomic prediction models to predict metabolite traits in another oat panel consisting of more advanced breeding lines. By integrating metabolite structures or biosynthetic pathway information into genomic prediction models, prediction accuracy of metabolites measured by liquid chromatography–mass spectrometry increased, but prediction accuracy of metabolites measured by gas chromatography–mass spectrometry were not improved.

## 12 GENEBANK GENOMICS DISENTANGLES AMARANTH TAXONOMY

The first step in plant breeding is to understand the morphological and genetic backgrounds of the plant material, which are both poor for the nutrient‐rich amaranth genus. Lin et al. (https://doi.org/10.1002/tpg2.20206) used a cost‐effective sequencing technique to investigate the genetic diversity among eight amaranth species in the WorldVeg gene bank. With these DNA markers, the passport information is updated for the misclassified amaranth accessions. In the aspect of morphology, dual‐purpose amaranth species have larger leaves than leaf amaranth species and flower later than grain amaranth species. This phenotypic divergence reflects the domestication of plants for different purposes. To accelerate amaranth breeding progress, the quantitative trait loci regulating days to flowering were mapped for grain amaranth complex and leaf amaranth collections. The candidate genes show great potential for marker‐assisted selection in amaranth breeding.

## 13 VARIANT INFORMATION ON GROWTH AND WOOD QUALITY TRAITS IN *EUCALYPTUS* IMPORTANT TOOL FOR BREEDING

Understanding the genetic basis of traits is an important prerequisite for breeding aimed at improving plant productivity. Tan and Ingvarsson (https://doi.org/10.1002/tpg2.20208) show that including information on variants that are important for controlling variation in growth and wood quality traits in *Eucalyptus* also provide important information for breeding. Including information on such genetic variants into genomic prediction models significantly increase the accuracy of predicted phenotypes. Also, including information on both additive and dominance effects are beneficial for improving genomic prediction, especially when dealing with breeding material derived from multiple species.

## 14 MICRORNA‐MEDIATED NETWORKS IN *BLETILLA STRIATA*

The molecular mechanisms related to the regulation of secondary metabolism have not been characterized in *Bletilla striata* possessing both ornamental and medicinal values. In this study, integrated analysis of RNA, small RNA and degradome sequencing data from three organs (leaf, root and tuber) of *B. striata* provided Ma et al. (https://doi.org/10.1002/tpg2.20210) with a comprehensive view of the microRNA (miRNA)‐mediated regulatory network. Organ‐specific expression patterns of the protein‐coding genes were analyzed. A total of 365 miRNAs were identified and subject to expression pattern analysis. A total of 1,142 miRNA—target pairs were validated for network construction. Some miRNA‐mediated regulatory pathways were indicated to be monocot‐specific. Our results lay a solid basis for in‐depth studies on the regulatory mechanisms underlying the production of the medicinal ingredients in *B. striata*.

## 15 PATH TO PENNYCRESS DOMESTICATION

Pennycress is a brand‐new winter oilseed crop in the U.S. landscape undergoing direct domestication. However, the genetic control of various secondary domestication syndrome traits including increased seed size and oil content is not clearly understood. Tandukar et al. (https://doi.org/10.1002/tpg2.20211) discovered fifty‐nine total marker trait associations revealing genomic regions controlling six different seed characteristic traits via genome‐wide association studies. A global pennycress association mapping panel was put together, and 33,606 high‐quality SNPs derived via genotyping‐by‐sequencing was leveraged to understand the inherent population structure and linkage disequilibrium decay within the population. Reliability estimates for important yield component traits like thousand grain weight, seed area, length, width, protein, and oil content have been reported for the first time. These phenotypic and genetic data will provide a solid foundation for continuing research on the genetic improvement of seed size and oil quality in pennycress.

## 16 NEW TOOL TO FIGHT WHEAT STRIPE RUST

More virulent and aggressive races of the wheat stripe rust pathogen, *Puccinia striiformis* f. sp. *tritici* that appeared in the year 2000 are now established in many countries, threatening global wheat production. Therefore, there is an urgent need to identify and deploy durable resistance genes against these new races. Dang et al. (https://doi.org/10.1002/tpg2.20212) report in this study the precise mapping of the adult plant resistance gene *Yr78*, which is effective against the new races. The gene was mapped to a small interval on the short arm of chromosome 6B completely linked to the nucleolar organizing region. Using gene‐sequencing data from multiple wheat genomes, we identified a group of molecular markers linked to *Yr78* that suggest that this gene originated in the ancient Spelt wheats. This result indicates that *Yr78* has been protecting wheat from stripe rust for hundreds of years. We developed two diagnostic markers tightly linked to this gene that will be useful to accelerate the deployment of *Yr78* in wheat breeding programs and reduce yield losses caused by this pathogen.

